# Lest Death Do Them Part: Protection of Older Women From Intimate Partner Injury and Fatality

**DOI:** 10.1093/geroni/igaa057.2457

**Published:** 2020-12-16

**Authors:** Janet Wilson, Lazelle Benefield

## Abstract

Protecting and preventing older women from fatal and near fatal violence by current or former intimate partners are public health and international human rights concerns. Older women are most often killed (“femicide”), injured in the home by a spouse, partner, x-partner, or family member. Older women experience physical, psychological, sexual abuse, neglect, and financial exploitation. Wherein there is more research on risk factors, study of protections lags behind. Through three very different studies, risks and protections will be explored with national and international policy implications for each.

